# Observations on the Present and the Future of Hip Surgery

**DOI:** 10.3390/jcm12103464

**Published:** 2023-05-14

**Authors:** Alberto Di Martino

**Affiliations:** 1 1st Orthopedic Department, IRCCS Istituto Ortopedico Rizzoli, 40136 Bologna, Italy; albertocorrado.dimartino@ior.it; 2 Department of Biomedical and Neurimotor Sciences—DIBINEM, University of Bologna, 40136 Bologna, Italy; 3 Sidney Kimmel Medical College, Thomas Jefferson University, Philadelphia, PA 19107, USA

Each period in history has its own peculiar fashions and trends, and contemporary research on hip surgery is no exception. In the last 20 years, major efforts have been directed towards the study of the effects of implant modularity and tribology [1,2,3] and the use of big-headed implants on durability and the risk of failure [4]. However, it is now well known that while most of the choices made by hip surgeons and implant developers are associated with successful improvements, some of these have been connected to specific complications and implant failure that required revision surgery. Understanding the positive and also the negative evolution of orthopedic implants, and deepening the biological basis of implant osteointegration, have gradually improved the results of total hip arthroplasty (THA) surgery. 

The present and the future of hip surgery include several different topics, mainly research on minimal invasiveness and enhanced recovery after surgery protocols, newer technologies to improve the outcome of hip replacements and, because of the increased number of primary implants, newer standardized approaches to revision THA surgery [5]. At present, hip reconstruction surgeons have the main aim of improving the postoperative recovery of THA patients, allowing them to return to full function and satisfactory daily living. Given the good outcomes of THA surgery, it was named “the surgery of the century” [6]. Hip surgeons promote the implementation of minimally invasive surgical approaches to the hip to minimize the trauma to the joint and soft tissues, and to allow early hospital discharge and return to daily activities [7]. Fast-track protocols require an unprecedented effort in terms of planning and preparation by doctors and all healthcare professionals. Dedicated anesthesiology protocols allow pain control and recovery, promoting early discharge [8]; patients can ambulate, use crutches and be discharged home in most cases on the same day as the surgery or the next day [9].

The implementation of minimally invasive surgery has allowed the support and extension of the indication of THA to younger patients for the first time, who require an early return to function and good aesthetic results [10], but it has also provided a unique opportunity to improve the quality of life in fragile elderly patients. In this sense, the direct anterior approach (DAA) has gained wide popularity in the last few years because of its intrinsic minimal operative trauma to the hip joint and because of the positive impact on implants’ survival, which is achieved independently from body habitus, age [10] and bony anatomy [11,12,13]. However, since the DAA has gained popularity, many surgeons have experienced its effectiveness in the management of hip diseases, either performing a mini-open preservative procedure for slipped capital femoral epiphysis [14] or performing neck reshaping for femoral–acetabular impingement [15]. Given the expertise developed by surgeons in this field, more complex anatomy and surgical cases are managed through the extended anterior approach, above all when the pathology affects the anterior aspect of the hip, as in the case of flexion contractures of the hips or calcifications in the context of the psoas muscle or anterior to the capsulae [16,17].

As our ability to develop more sophisticated technologies has increased, substantial efforts have been directed towards the implementation of machines and software to support the skills of orthopedic surgeons [18]. A lot of current research is related to the implementation of newer technologies to improve patient-specific implant positioning accuracy, both preoperatively and intraoperatively. In the preoperative setting, correct templating is still required, and this can be performed through 2D imaging software and sometimes through 3D reconstruction [19]. Three-dimensional visualization is crucial in complex cases, and it is the base for the design of custom implants, nowadays used most in revision THA surgery [20].

Revision THA surgery is expected to grow in the near future because of the very high number of primary THA cases each year worldwide [21]. It is a kind of surgery that requires dedicated instrumentation and surgeon expertise, and an acceptance of the risk of failure and complications, which are classically increased in this patient population. An accurate knowledge of the causes and modes of failure of THA implants is required, and this may be enriched by the constant update of registries that monitor the survival of hip implants worldwide. Among the most common causes of failure, dislocation and aseptic and septic loosening are among the most frequently reported [22]. 

To improve the chance of the integration of implants through bone ingrowth at the bone–implant interface, newer biomaterials have been introduced into the market to promote the bone integration of the implant surface. Nowadays, tantalum and trabecular titanium are the technological bases for the design of modern implants used in revision cases. Modern technologies allow us to develop and produce personalized implants that are aimed at the filling of the bone defects occurring after the failure of THA implants or during implant removal [23].

While at present, computer-assisted surgery and navigation are the most promising methods for implant placement accuracy, proper patient study and preparation are quintessential. Artificial intelligence-based programs aimed at the production of personalized implants may, in the future, ease the surgery and bone stock reconstruction of patients [24]. 

As technology is changing our lifestyle and many aspects of human relationships, we are confident that in the future, scientific progress will be able to reduce human error and improve the durability of hip implants. However, despite these advancements, doctors should never forget both the human being behind the technology and the patient behind the radiography and implant.
